# COVID-19 and Food: Challenges and Research Needs

**DOI:** 10.3389/fnut.2020.598913

**Published:** 2020-12-03

**Authors:** Dietrich Knorr, Chor-San H. Khoo

**Affiliations:** ^1^Technische Universität Berlin, Berlin, Germany; ^2^International Life Sciences Institute of North America, Washington, DC, United States

**Keywords:** food, nutrition and COVID-19, food and water safety, food and water security, COVID-19 and supply chain, technology and pandemic

## Abstract

The paper highlights several food and nutrition-related challenges encountered during the COVID-19 pandemic, including food and water safety, supply chain disruptions, food and water insecurity, consumer and food behavior, malnutrition and nutrient intakes, food surveillance technology, as well as potential post-COVID-19 strategies. Its main objective is to stimulate robust scientific discussions on existing research gaps and to develop long-term “exit strategies” to prepare for future pandemics.

## Introduction

At the turn of this century, there were speculations whether events of the “roaring 1920s” would return a century later. What does return is another zoonotic pandemic, COVID-19, potentially as destructive as the “Spanish Flu” pandemic of 1918–1919 that was caused by the H1N1 virus with genes of avian origin. The 1918 flu affected one-third of the world's population and claimed over 50 million lives (1, 2). The current COVID-19 pandemic, believed to begin in winter of 2019 in Wuhan, China, is traced to a novel corona virus (SARS-CoV-2) with animal origins. By May 6, 2020, cases of COVID-19 infections have spread to over 170 counties, with over 3.6 million reported infected cases (3–6). By August 6, the global infected cases approached 20 million with almost 713,000 deaths, and warning of impending second waves.

With the uncertainty of a vaccine in the foreseeable future, mitigation strategies have been put in place that focus on social distancing, facial covering, frequent handwashing, stay at home orders, working from homes, and testing taken on by public health priorities. These efforts lead to drastic reduction in the workforce, travel, business, economy, food supplies, and overload the healthcare system. There are also reports of changes in consumers' food behaviors—purchase, choices, preparation, and eating patterns.

As the COVID-19 pandemic continues, there is a need to question whether the current food system is resilient to withstand a prolonged and pervasive global pandemic. The twenty-first century food system is intrinsically complex and dynamic with many interrelated and interdependent essential components working in tandem to ensure a resilient food supply. Any disruption to one or more of these connections will affect the stability of the global food supply. Huff et al. (7) illustrated the interrelated impact of a pandemic on multiple fronts—communication and transportation systems, product and agricultural supplies—all of which lead to food and material shortages. A review of the impact of the coronavirus on agriculture (8) suggests the pandemic affects mainly on supply, demand, labor, food safety, and security.

## Objectives

In this paper we highlight several challenges that the food and nutrition communities encounter during the COVID-19 pandemic that limit definitive actions during and post pandemic. We hope that raising these broad issues will stimulate future dialogues and robust scientific discussions on current research gaps, future research needs among food and nutrition related communities for developing a foundational framework for collaborative solutions and partnership to build a resilient food and nutrition system in readiness for future pandemic disruptions.

## Challenges and Future Needs

### Food and Water Safety

#### Viral Survivability

According to WHO, FAO, and the European Commission (4, 9) it is highly unlikely that SARS-CoV-2 virus can be transmitted from food or food packaging. So far, the evidence regarding person-to-person transmission of the illness remains under investigation. Limited but emerging studies conducted under laboratory conditions have reported the persistence of SARS-CoV-2 and SARS-CoV-1 on various types of surfaces (10, 11). These studies implied that the survival of the virus depends on the type and condition of the surfaces (paper, steel, copper), humidity, initial virus concentration, environmental temperature, and time. Due to different methods used to measure virus presence (PCR, metagenomic), it remains challenging to provide definitive actions. Recently, Anelich et al. review the hazard-risk related to COVID-19 in foods and conclude that the overall potential risk of acquiring COVID-19 from contaminated food or packaging is low (12).

A similar situation exists for drinking water (9) regarding virus persistence in drinking water. Though there is a possibility of survival, so far, no SARS-CoV-2 virus has been detected in tap water. This is also the basis for recommendations for proper hand hygiene by all relevant governmental organizations.

#### Future Needs

1. Study to understand the factors and conditions that impact the survivability of SARS-CoV-2 in food, drinking water, and food surfaces.
2. More reliable and accurate data needed on SARS-CoV-2 persistence in different types of food products prepared under different processing/preparation/storage techniques.
3. Develop a solid foundation of good hygiene practices and implement effective food safety management systems along the food chain.
4. Innovating and better understanding and advanced technology platform for virus inactivation on food and packaging materials, and to understand the principles of mode of actions.
5. Advanced approaches for viral elimination in drinking water supplies.

### Supply Chain, Trade, and Delivery Disruption

International and national food suppliers and customers are severely impacted by national shutdowns and restrictions, affecting jobs, economic growth, economy, growth, and mobility (13, 14). The restriction on guest workers to work on farms and manufacturing plants have led to widespread disruption in planting and harvesting of food, triggering unintended consequences of unnecessary food losses and wastes. With business shutdowns and unemployment increase, food insecurity will increase the risk of poverty with the most vulnerable individuals being likely hit the hardest (14–16).

#### Future Needs

1. Explore a model of radical change of existing supply chain systems to one that will increase national self-sufficiency, broaden supply chain, and incorporate rapid tracking and tracing systems.
2. Explore a new and more targeted public educational initiative that enhances public understanding of food value, food processing and packaging approaches, and food safety during a prolonged pandemic.
3. Explore paths to deliver fast, credible, and clear food information, healthy food practices, and food waste reduction.
4. Studies to understand the impact of home deliveries on nutritional quality and food safety of home-delivered foods associated with true foodborne pathogens.
5. Explore packaging that is safe, resilient, sustainable, and cost effective in prolonged and pervasive pandemics.

### Food and Water Insecurity

The top three UN Sustainable Development Goals are no poverty, zero hunger, and good health and well-being. All three of these goals are most likely to be affected by the COVID-19 crisis (16, 17). The Global Report on Food Crises 2020 predicts that “conflict/insecurity, weather extremes, desert locust, economic shocks, and COVID-19 are expected to be key drivers of food insecurity” (18). Naidoo and Fisher (19) argue that COVID-19 is exposing the fragility of all the 17 Sustainable Development Goals, set by the United Nations and based on sustained economic growth and globalization, with all of them now being somehow threatened. A joint statement of FAO, IFAD, the World Bank, and WFP (20) states: “the pandemic is already affecting the entire food systems.” This stresses the urgent need to develop an approach that integrates both the food system and the value chain (21, 22). This requires a radical change to current R&D practices, to one that also must model in the challenges of our food systems, including accessibility, availability, and affordability. The new World Food Program (23) figures suggest that an additional 130 million lives and livelihoods will be at risk indicating that due to the COVID-19 pandemic, acute hunger will almost double by the end of 2020. The number of people displaced globally is estimated at 71 million and 370 million children with no access to school lunches due to national lockdowns (24). A current USDA assessment reports that the number of people who considered food as not secure is estimated at almost 20% of the total population of the 76 low- and middle-income countries examined (25). As the recommendation for COVID-19 and food safety emphasizes the need for frequent handwashing with soap and water, or the use of alcohol-based hand sanitizers (9, 26, 27), these resources are not available or accessible to the low- income and poor population, which are already hardest hit with disease comorbidities. Thus, these guidelines become unachievable and obsolete to these populations.

#### Future Needs

1. Stress the importance of protective hygiene and protection measures for humans.
2. Improvement in current efforts to increase in the availability and accessibility of safe food and water to poor populations, and to prevent fraud and misbranding.
3. Develop interdisciplinary food systems and value chain integration approaches.
4. Increase efforts to explore new ways for food and water recovery and generation.

### Consumer Food Behavior

COVID-19 is changing where, how, and what consumers eat. There is report of more online shopping, fewer grocery trips, but more food purchased per trip, and more snacks and ready-to-eat meals. In a survey study of 1,000 adults in the United States on their food behavior and practices during COVID-19 (5), 80% of the respondents indicated they were somewhat confident that the food they were buying was safe; 50% of the respondents indicated that they had reduced in-person shopping, 40% indicated that they bought more packaged foods, and 47% indicated that they were eating more home cooked meals. In the US household, food spending increased due to stockpiling during the COVID-19 pandemic as reported by Baker et al. (28). A silver lining has emerged amidst the recent pandemic. There is a growing appreciation for food value, food safety, and healthy food intakes, as well as an awareness of the need to reduce household food wastes.

#### Future Needs

1. Understanding the changes in consumers' food in an environment in response to mitigation efforts.
2. Understanding overall impact of pandemic-related circumstances on consumer food and nutrient intakes, and dietary patterns.
3. Study the impact of increased home food delivery on households and individual nutritional and health status (fitness, weight, blood pressure, risk factors) during a pandemic, etc.).
4. Food safety habits and hygiene practices regarding consumers, and food processing, handling, and preparation.
5. Understanding dynamics and psychology of food choices and intakes during and post pandemics and effects on long-term food behavior.
6. Study of the impact of COVID-19 on consumer food home preparation and food waste reduction.

### Diet and Nutrition

Recent data suggest that in humans, SARRS-CoV-2 affects the respiratory systems as well as other organs (29) indicating the high complexity of the human immune system to protect against invading pathogens or foreign material. Though nutrients such as copper, folate, iron, selenium, zinc, vitamins A, B6, B12, C, and D play critical roles in supporting the immune system, it is not likely that the viral infection can be modified acutely by a specific nutrient or food intervention. At different phases of the infection or disease progression, different and multiple nutrients or food may be involved. Currently, there is no food or nutrition intervention known to stop COVID-19. However, a healthy and diverse diet and nutrition can support and modulate immune responses to viral infections (29). The interaction between gut microbial diversity and COVID-19 especially in elderly has been indicated (30), and the importance of oral microbiota and good oral hygiene has also been stressed (31). A well functioning immune system is critical to a robust response to any pathogenic infections (32). Current dietary guidelines to follow a healthy diet has been recommended to be the best support to the immune system including possibly delaying immunosenescence or providing robust response to pathogenic infection (32, 33).

#### Future Needs

1. There is a need to understand the link between nutritional status and COVID-19, and the impact of nutritional health in recoveries in different population groups.
2. Research to address the potential impact of nutritional status on morbidity and mortality from SARS-CoV-2.
3. Close the gap in knowledge in the modulating role food and nutrients play in COVID-19.
4. Develop current healthy guidelines and eating pattern recommendations applicable for pragmatic adherence during a pandemic, e.g., management of COVID-19.
5. Explore adaptation of traditional care pathways to allow temporary mechanisms for pragmatic and safe decision making for public on behavioral changes in nutrition to embed nutritional care into COVID-19 health care practice.
6. Initiate long-term studies on relationships between human diets and immune system related to SARS viral effects
7. Increase efforts to improve knowledge relating protective effects of human microbiota.

### Food Surveillance and Technologies

As COVID-19 affects many fronts simultaneously, food surveillance measures of the pandemic impact needs to be monitored from multiple different perspectives to afford a wholistic overview. Jaykus and Hoelzer (34) proposed that food surveillance measures should include consumer-eating habits, healthcare- seeking behavior, healthcare delivery changes (telemedicine), healthcare delivery capacity, public health laboratory testing, and epidemiological capacities. The use of technologies to expedite and implement mitigation strategies is central to assess mitigation success, weakness, and impact in a pandemic. Recently, Tonby (35) reported six technologies that have helped several Asian governments and businesses to shape the region's early response to COVID-19 safeguarding disruptions in health and livelihood. These technologies, which could be used as general guidance worldwide, enable rapid track–trace–test quarantine cycles, monitor and manage surges in health care delivery capacity, enable rapid dissemination of pandemic information to the public, digitalizing products and services, enabling remote working and learning (35).

#### Future Needs

1. Identification of critical factors to provide wholistic models for future food surveillance in a pandemic.
2. Identification of effective technologies that can provide effective public information strategies.

### Exit Strategy

From 1347 to 1351, the Black Death killed between 30 and 60% of all Europeans. Recent work on this past pandemic revealed that social and economic inequalities already then shaped its course (36). More recently, food economists have been warning the global “just-in time” food delivery chains were inherently at risk of failing (37) as the current disruption within the food chain demonstrates. Assessing exit strategies across Europe using mobility data, Ruktannonchai et al. (38) warned against ending stringent national and successful interventions prematurely and found that appropriate coordination across the continent could aid in eliminating COVID-19 transmission. In addition, the importance and need for behavioral changes has been stressed (38, 39). According to a McKinsey & Company report (35), technology can make a difference on how business and society respond to risks of a global pandemic.

#### Future Needs

1. Development of tools for rapid and transparent information and for effective surveillance of the public.
2. Provision of clear food safety and hygiene requirements for every step along the food chain.
3. Establishment of strict and global traceability tools for food and food constituents.
4. Creation of food safety and hygiene regulations for all food-handling personnel.
5. Initiation of local/regional storage facilities for food staples and drinking water.
6. Increasing local/regional self-sufficiency for food and water supplies.
7. Designing alternative concepts to current “just in time” for availability practices.
8. Assurance of continuous feeding programs for poor populations during school closures and lockdown with nutritious food.
9. Provision of local/regional recommendations for shelf stable food storage.

## Conclusion

We urge the nutrition and food-related scientific communities to develop ideas, concepts, position papers, research, and educational activities on how to address challenges arising from COVID-19 pandemic, and solutions for recovery from the current crisis. In addition, we encourage the scientific communities to anticipate needed food and nutrition priorities for future pandemics, and to build a more resilient food system that better integrate critical factors such as the food supply chain, nutritional security, healthy food accessibility, education, and communication on food safety and preparation in the event of long quarantines. Ensuring confidence in the food supply through enhanced public education of food value, processing techniques, and food safety are critical elements. Developing long-term “exit strategies” and goals are necessary to prepare for future pandemics. Lawton (40) warned that “amid the pandemic, a second epidemic of preliminary, unverified and misinterpreted research has broken out.” The race to publish underscores the importance of having an advisory body to protect and maintain data, and scientific quality, integrity, and transparency. Subsequently, there is a need to form a global food, nutrition, and related science advisory body to prepare and advise for future pandemics as also suggested by Loeb and Gil (41). Recommendations by the advising scientific body will need also to be shared and adopted by decision makers to enable successful and sustained implementations of programs.

## Author Contributions

All authors listed have made a substantial, direct and intellectual contribution to the work, and approved it for publication.

## Conflict of Interest

The authors declare that the research was conducted in the absence of any commercial or financial relationships that could be construed as a potential conflict of interest.
